# Lack of Effect of a Single Injection of Human C-Reactive Protein on Murine Lupus or Nephrotoxic Nephritis

**DOI:** 10.1002/art.27232

**Published:** 2009-12-28

**Authors:** Francesco Carlucci, H Terence Cook, Abhilok Garg, Mark B Pepys, Marina Botto

**Affiliations:** 1 Imperial College LondonLondon, UK; 2 University College LondonLondon, UK

## Abstract

**Objective:**

It has been reported that a single dose of human C-reactive protein (CRP) can prevent and reverse the renal damage in murine models of spontaneous lupus, as well as the rapid-onset immune complex disease induced in the accelerated nephrotoxic nephritis (ANTN) model. This study was undertaken to attempt to replicate these observations using a highly purified and fully characterized human CRP preparation.

**Methods:**

(NZB × NZW)F_1_ (NZB/NZW) mice were treated with a single 200-μg subcutaneous injection of CRP or control reagents either before disease onset at 4 months of age or when high-grade proteinuria was present at 7 months of age. Mice were monitored at least monthly for proteinuria and autoantibody levels. ANTN was induced by preimmunizing C57BL/6 mice with sheep IgG, followed 5 days later by injection of sheep anti-mouse glomerular basement membrane antibody and CRP or control reagents. Renal disease was assessed by regular urinalysis and histologic evaluation.

**Results:**

CRP treatment of NZB/NZW mice, either early or late in the disease, had no effect on proteinuria, autoantibody titers, or survival. CRP administration did not reduce renal injury or alter disease in the ANTN model. Human serum amyloid P component, a pentraxin protein that is very closely related to CRP, similarly had no effect.

**Conclusion:**

Our completely negative observations do not confirm that human CRP has reproducible antiinflammatory or immunomodulatory effects in these murine models, nor do they support the suggestion that CRP might be useful for therapy of lupus or immune complex–mediated nephritis.

C-reactive protein (CRP), a member of the pentraxin family of proteins, is the classic human acute-phase protein (1). It binds, in a calcium-dependent manner, with highest affinity to phosphocholine residues, and although it does not bind to healthy living cells, its autologous ligands include the membranes of damaged and dead cells and small nuclear RNP particles (2), which are important autoantigens in systemic lupus erythematosus (SLE). CRP does not bind to chromatin under physiologic conditions, whereas the closely related human pentraxin protein, serum amyloid P component (SAP), avidly binds to DNA and nuclear chromatin (3). Bound CRP potently activates complement via the classical pathway and can thereby engage all of its proinflammatory and opsonic functions (1).

Based on the binding of CRP to potential autoantigens and its capacity to precipitate or agglutinate them and to activate complement, investigators at our laboratory originally proposed that CRP might function to safely scavenge cellular debris (4). Absent or inappropriately modest acute-phase responses to disease activity in human and murine SLE have long been recognized, and it was suggested that failure to mount an adequate acute-phase response might predispose to autoantibody formation and thus to SLE (5). The reports by du Clos and colleagues and Rodriguez and colleagues describing immunosuppressive effects of single injections of human CRP into mice with either spontaneous or induced immunologic disease (6–9) were therefore of considerable interest. In the present investigation we attempted to reproduce these findings.

## MATERIALS AND METHODS

### Animals

Female wild-type C57BL/6 and (NZB × NZW)F_1_ (NZB/NZW) mice (Harlan, Blackthorn, UK) were kept under specific pathogen–free conditions and handled throughout according to institutional and national legal guidelines.

### Reagents

Intact, fully functional native CRP was isolated at >99% purity from malignant effusion fluids by DEAE anion exchange followed by calcium-dependent binding to immobilized phosphoethanolamine and elution with free phosphocholine. The isolation process is described in detail in the supplementary text, available in the online version of this article at http://www3.interscience.wiley.com/journal/76509746/home. Phosphocholine was dissociated by addition of EDTA to achieve a concentration of 10 m*M* and removed by buffer exchange into 10 m*M* Tris, 140 m*M* NaCl (pH 8.0). After addition of CaCl_2_ to achieve a concentration of 2 m*M*, the CRP was concentrated to ∼4 gm/liter and stored frozen at −80°C. It was diluted for in vivo use with 10 m*M* Tris, 140 m*M* NaCl, and 2 m*M* CaCl_2_ (pH 8.0). The purity of the CRP preparation was analyzed by sodium dodecyl sulfate–polyacrylamide gel electrophoresis (8–18% gradient) with 50 μg sample loading, Brilliant blue R-350 stain, and a single-band sensitivity limit of 0.1 μg. Molecular integrity and native conformation were confirmed by size-exclusion chromatography, sedimentation equilibrium ultracentrifugation, x-ray and neutron solution scattering, electrospray ionization mass spectrometry, crystallization, and solution of the x-ray 3-dimensional structure. CRP concentration was measured in both Roche MIRA (Ramsey, MN) and Dade-Behring (Milton Keynes, UK) BNII latex-enhanced immunoassays. Binding to phosphocholine was measured in solution by isothermal titration calorimetry, C1q binding was demonstrated using enzyme-linked immunosorbent assay (ELISA) and surface plasmon resonance assay, and classical pathway C activation was monitored by C3 binding and cleavage with pneumococcal C–polysaccharide as ligand. These exhaustive tests confirmed that the material was pure, not aggregated, and retained full native structure, immunochemical integrity, and functional properties.

The endotoxin content of the CRP preparation, determined by the *Limulus* kinetic turbidimetric method (Cambrex, Nottingham, UK) according to European Pharmacopoeia monograph 2.6.14 (2005), was 0.9080 endotoxin units (EU)/mg (10). There was no acute-phase response of either mouse serum amyloid A protein or SAP, the two most sensitive mouse acute-phase proteins, to injection of this CRP into mice, nor did it stimulate cytokine production by mouse macrophages in vitro (10). Human SAP was isolated at >99% purity as previously described (11). High-purity human albumin (serum fraction V) was purchased from Calbiochem (Nottingham, UK). These proteins were diluted in 10 m*M* Tris, 140 m*M* NaCl (pH 8.0) for in vivo use.

### Induction of accelerated nephrotoxic nephritis (ANTN) and CRP treatment

Female 8–10-week-old C57BL/6 mice received 200 μg of purified sheep IgG (Sigma, Poole, UK) in complete Freund's adjuvant (Sigma) by intraperitoneal injection 5 days before intravenous injection of 5 mg of sheep nephrotoxic serum (12). The endotoxin content of the nephrotoxic serum preparation was <0.1 EU/mg. Mice (10 per group) received 200μg CRP or, as controls, 200 μg albumin, 200 μg SAP, or Tris–saline vehicle alone, by subcutaneous injection given at the same time as nephrotoxic serum injection. Urinalysis with Hema-Combistix (Bayer, Newbury, UK) was performed on alternate days. Hematuria was scored as 0 (negative), 0.5 (trace), 1 (+), 2 (2+), or 3 (3+). Proteinuria was scored as 0/trace (<0.30 gm/liter), 1+(≥0.30 gm/liter), 2+ (≥1 gm/liter), 3+ (≥3 gm/liter), or 4+ (≥20 gm/liter). Complete 24-hour urine collections were obtained immediately before the mice were killed 10 days after nephrotoxic serum injection. Urine albumin concentrations were measured by radial immunodiffusion using rabbit anti-mouse albumin antibody (Biogenesis, Poole, UK) and purified mouse albumin standards (Sigma).

### CRP administration to NZB/NZW mice

Female 4-month-old NZB/NZW mice were randomly allocated to 4 experimental groups of 10–20 mice. Different groups received a single subcutaneous injection of 200 μg CRP, 200 μg albumin, 200 μg SAP, or vehicle alone. Blood and 24-hour urine collections were obtained monthly. Proteinuria was assessed with Hema-Combistix and autoantibodies titrated as described below. Animals were killed when they developed signs of renal failure and were recorded as deaths in the survival curves. Separate cohorts of female 7-month-old NZB/NZW mice with 4+ proteinuria were injected subcutaneously with 200 μg CRP, 200 μg albumin, or vehicle alone. Proteinuria was then monitored daily for 7 days and weekly for a further 3 weeks. Animals were killed if they developed signs of renal failure with weight loss.

### Renal histology

Kidneys were fixed in Bouin's solution, embedded in paraffin, and sections were stained with Periodic acid–Schiff reagent. Glomerular cellularity was scored under blinded conditions, as grade 0 (normal), grade 1 (hypercellularity involving >50% of the glomerular tuft in 25–50% of glomeruli), grade 2 (hypercellularity involving >50% of the glomerular tuft in 50–75% of glomeruli), grade 3 (hypercellularity involving >75% of glomeruli or crescents in >25% of glomeruli), or grade 4 (severe proliferative glomerulonephritis in >90% of glomeruli). Crescent formation, periglomerular fibrosis, and tubular changes were also recorded.

### Assays for anti–single-stranded DNA (anti-ssDNA) and anti–double-stranded DNA (anti-dsDNA) antibodies

Anti-ssDNA IgG antibodies were measured by ELISA as previously described (13).

### Statistical analysis

Kaplan-Meier survival curves were compared by log rank test. Kruskal-Wallis nonparametric analysis of variance was applied throughout. *P* values less than 0.05 were considered significant. Statistical tests were performed with GraphPad Prism, version 3.0 (GraphPad Software, San Diego, CA).

## RESULTS

### Effect of human CRP treatment on accelerated nephrotoxic nephritis

**Figure 1:**
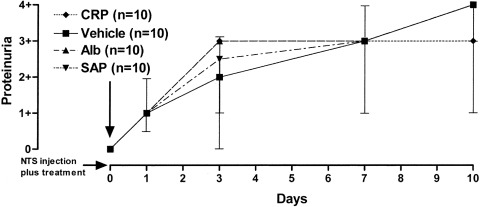
Effect of treatment with C-reactive protein (CRP), albumin (Alb), serum amyloid P (SAP), or vehicle alone on development of proteinuria in C57BL/6 mice with nephrotoxic nephritis. Values are the median and range in 10 mice per treatment group. NTS = nephrotoxic serum.

There was no delay in onset or reduction of proteinuria following nephrotoxic serum administration in the mice treated with CRP compared with those that received control reagents (Figure 1). On day 10, all of the mice had ≥3+ proteinuria and signs of renal impairment. All animals were therefore killed at this time and albuminuria, hematuria, and renal histology were assessed. Consistent with the urinalysis strip results, there was no significant difference between the 4 groups in albumin concentrations in the 24-hour urine collections obtained the day before death (Table 1). Histologic analysis with light microscopy also showed the same degree of inflammation in all groups, with no differences in glomerular cellularity, crescent formation, periglomerular fibrosis, or tubular changes (Table 1).

**Table 1:** Effect of CRP treatment on albuminuria, hematuria, and glomerulonephritis in C57BL/6 mice with accelerated nephrotoxic nephritis*

	Treatment			
	
	CRP (n = 10)	Vehicle (n = 10)	Albumin (n = 10)	SAP (n = 10)
Albuminuria, mg/liter	177 (61–381)	177 (81–500)	177 (81–381)	202 (106–293)
Hematuria grade	1 (0–2)	0.5 (0–2)	2 (0–3)	2 (0–3)
Glomerulonephritis grade	3 (2–4)	3 (1–3)	2 (1–3)	3 (1–4)

* Values are the median (range). CRP = C-reactive protein; SAP = serum amyloid P.

### Effect of CRP treatment on autoimmune disease in NZB/NZW mice

**Figure 2:**
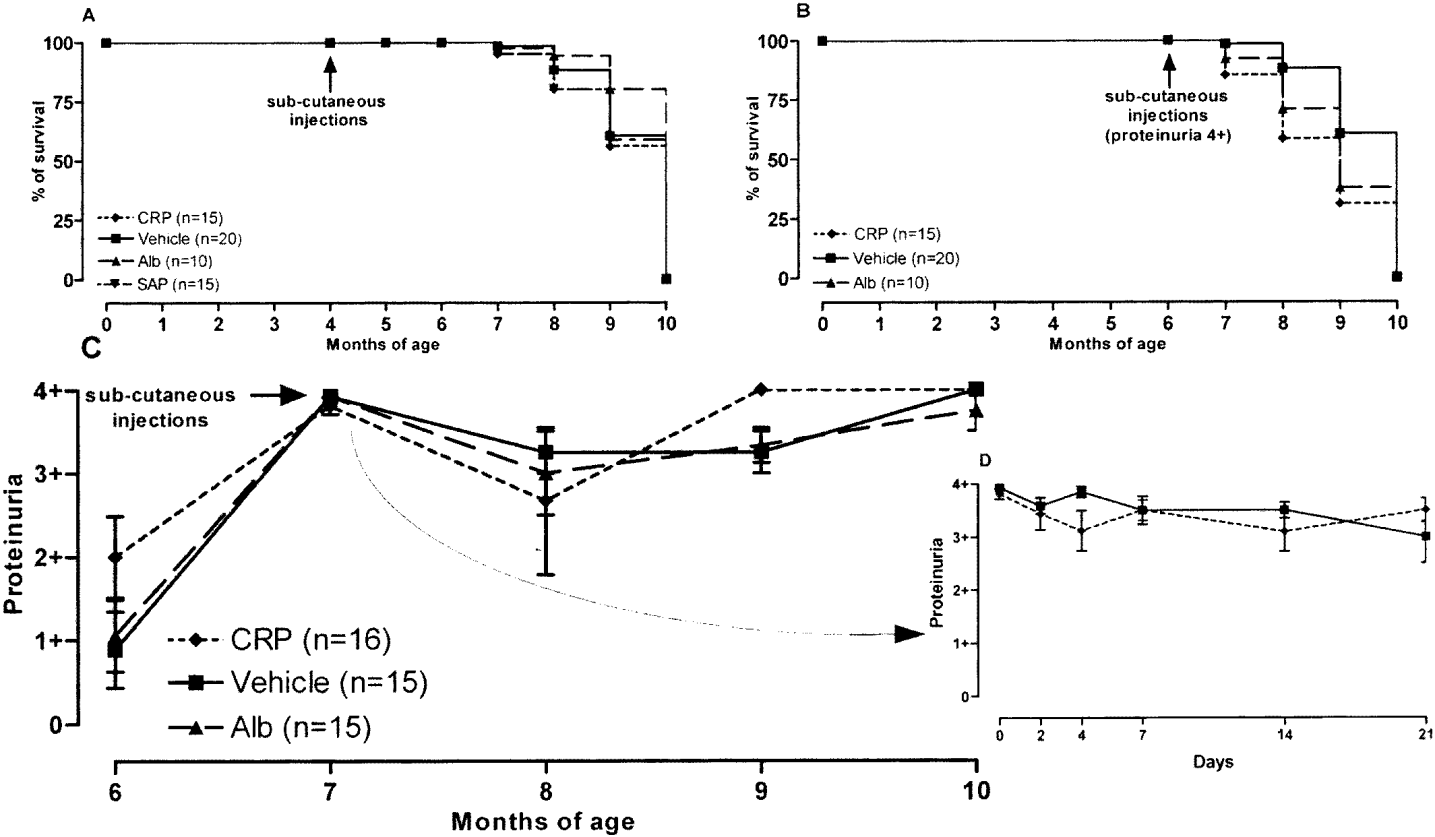
Effect of CRP on onset and progression of disease in female (NZB × NZW)F_1_ mice. **A** and **B,** Survival of animals treated with CRP, albumin, SAP, or vehicle before **(A)** and after **(B)** the onset of renal disease as indicated by 4+ proteinuria. **C** and **D,** Proteinuria measurements obtained monthly **(C)** and more frequently during the first 3 weeks **(D)** in the groups injected at 7 months of age. Values are the median and range. See Figure 1 for definitions.

Injection of a single subcutaneous dose of 200 μg CRP in NZB/NZW mice at 4 months of age, when circulating autoantibodies were present only at low titer and there was no proteinuria, had no effect on the progression of autoimmune disease. Proteinuria appeared and progressed, and titers of anti-ssDNA and anti-dsDNA antibodies increased, at the same time and with the same tempo as in the control groups that received 200 μg human albumin, 200 μg SAP, or saline alone. The median survival time was 10 months in all groups (*P* = 0.57) (Figure 2A).

Similarly, when 200 μg CRP, 200 μg human albumin, or saline alone was injected into NZB/NZW mice at 7 months of age, when all the mice had developed high-grade (4+) proteinuria, there was no difference in median survival between the groups (Figure 2B). Close monitoring of proteinuria during the first 3 weeks following the subcutaneous injections, as well as monthly urinalyses, showed no sign of any CRP-mediated renal protection (Figures 2C and D). Furthermore, the CRP treatment did not alter serum levels of anti-ssDNA or anti-dsDNA IgG antibodies during the disease course (data not shown).

## DISCUSSION

Following our group's original suggestion that a function of human CRP might be to safely scavenge potential autoantigens (4, 5), the reports of immunomodulatory effects of injections of human CRP and of transgenic expression of human CRP in murine models of SLE-like autoimmune disease and nephrotoxic nephritis were very intriguing (6–9). These studies have evoked speculation about a possible therapeutic role for CRP in SLE, and it has been suggested that CRP exerts its proposed long-lasting protective effects by inducing interleukin-10 through engagement of Fcγ receptor I on macrophages (9).

We have been cautious about these surprising results for two important reasons. First, injected human CRP is very rapidly cleared in mice, with a half-life in the circulation of ∼3 hours (14), and is completely catabolized in the liver by hepatocytes (15). Either the CRP itself, or a contaminant present in the preparations used, would have to exert extraordinarily potent effects to down-regulate both the severe progressive life-long autoimmune disease of NZB/NZW and MRL-*lpr/lpr* mice and the aggressive tissue-damaging pathology of nephrotoxic nephritis. Second, although patients with SLE do not mount a major acute-phase response of CRP to progression and exacerbations of their disease, they do respond to intercurrent microbial infections with vigorous acute-phase responses and, apparently appropriate, high CRP concentrations (5). These episodes of greatly increased CRP production, which persist as long as the infection is not controlled and eliminated, have no detectable antiinflammatory or immunosuppressive effects. Indeed, intercurrent infection in SLE is well recognized as a potent trigger of relapse and exacerbation of the autoimmune disease process.

We therefore sought to reproduce the beneficial effects of single injections of human CRP in 2 of the models in which these effects have been reported. We used a single human CRP preparation that was isolated at very high purity from pooled malignant effusion fluids and exhaustively validated as the fully structurally and functionally intact native protein with no significant contamination by other human proteins or bacterial endotoxin. We controlled for specificity of any possible effects by treating different control groups with human serum albumin and with human SAP, the other pentraxin protein that is very closely similar to, but functionally distinct from, CRP. SAP is of particular interest in this context as it avidly binds to DNA and nuclear chromatin, which are the principal targets of the pathogenic autoantibodies in SLE.

Our uniformly negative results in both models do not reproduce previous observations of immunosuppressive and antiinflammatory effects of human CRP in mice and do not support the idea that either human CRP or SAP has potent or significant properties of this type. We are unable to explain these different sets of findings and have no information on which to base further speculation. We believe, however, that the rigorous and comprehensive characterization of our CRP preparation coupled with the scale of our experiments and the consistency of the findings across experimental and control groups strongly support the robustness of the present negative results.
