# Supplementary material for: Lack of Effect of a Single Injection of Human C-Reactive Protein on Murine Lupus or Nephrotoxic Nephritis
Source: Arthritis Rheum. 2009 Dec 28;62(1):245–9. doi: 10.1002/art.27232 (PMC3625741; doi:10.1002/art.27232)
Supplement: Supplementary file 1 [file art0062-0245-SD1.doc]

The present CRP was isolated as follows. Effusion fluids removed from the pleural and abdominal cavities of patients with confirmed metastatic malignancies in order to provide symptomatic relief were collected and retained instead of being discarded. All patients had a histological or cytological diagnosis and none had any infectious disease. All were negative by second generation FDA approved testing for HBsAg, anti-HIV-1 and 2, and HCV. Sodium azide to 0.1% w/v was added to all fluids on collection and they were then stored for up to 2 weeks at 4°C. After clarification to remove clot and cell debris 5% v/v of 0.2M EDTA, pH 7.0, was added to the pooled fluids and they were then mixed with DEAE Sephadex which had been swollen in distilled water containing 0.1% w/v sodium azide. Volumes of 125 l of pooled fluids were absorbed with 25 l swollen volume of DEAE for 1 hour at room temperature, and this was repeated up to four times with the same batch of DEAE. The DEAE was then washed with 3 lots of 25 l of 10 mM Tris, 140 mM NaCl, 10 mM EDTA, 0.1% w/v NaN_3_, pH 8.0, before eluting the bound proteins with 4 lots of 25 l of 2 M NaCl. Immediately after collection of each batch of eluate, 2.5 l of concentrated Tris, CaCl_2_, sodium azide solution was added to each 25 l volume to yield a final concentration of 10 mM Tris, 2 mM CaCl_2_ and 0.1% w/v NaN_3_ at pH 8.0. The pooled eluates were then mixed with 2 l of Sepharose bearing covalently immobilised phosphoethanolamine. This affinity matrix was synthesised by carbodiimide coupling of phosphoethanolamine to carboxyhexyl-Sepharose at the maximal density available, precisely according to the manufacturer’s instructions (GE Healthcare). After one hour at room temperature to enable the CRP to bind to the immobilised phosphoethanolamine, the fluids were removed by filtration and the resin was washed with 10 mM Tris, 140 mM NaCl, 2 mM CaCl_2_, 0.1% w/v NaN_3_, pH 8.0 until no further protein eluted. The beads were then poured into a 10 cm diameter chromatography column and the bound CRP was eluted at 6 l per hour flow rate with 10 mM phosphocholine in solution in the same wash buffer. The pooled eluted CRP was dissociated from bound phosphocholine by addition of EDTA to a final concentration of 10 mM to chelate calcium and the protein was then concentrated on a Millipore Pellicon device with a 30,000 Da cut off membrane before buffer exchange against 10 volumes of 10 mM Tris, 140 mM NaCl, 0.1% w/v NaN_3_, pH 8.0 to remove all phosphocholine and EDTA. After harvesting the concentrated CRP, 1 M CaCl_2_ was added to provide a final calcium concentration of 2 mM. Typical CRP concentration in the pooled effusion fluids was ~20 mg/l and recovery at this stage was typically 15-18 mg/l to yield typical batches comprising 6-9 g of CRP at greater than 95% purity and usually around 98-99% pure. Further purification was then conducted precisely as previously detailed (1).

1. De Beer, F. C., and M. B. Pepys. 1982. Isolation of human C-reactive protein and serum amyloid P component*. J Immunol Method*s 50:17-*31.*
